# Supplementary material for: Transmission dynamics of Klebsiella pneumoniae in a neonatal intensive care unit in Zambia before and after an infection control bundle
Source: PLOS Glob Public Health. 2026 Feb 9;6(2):e0005965. doi: 10.1371/journal.pgph.0005965 (PMC12885268; doi:10.1371/journal.pgph.0005965)
Supplement: S1 Table — (PDF) [file pgph.0005965.s001.pdf]

Table S1: Reference sequence accessions for each ST

Sequence type	Reference accession
ST15	NZ_CP062475.1
ST101	NZ_CP102940.1
ST147	NZ_CP029582.1
ST307	NZ_CP158302.1
ST983	NZ_CP021165.1
ST985	NZ_CP086724.1
ST2004	NZ_CP103579.1
