# Supplementary figures and images for: Transmission dynamics of Klebsiella pneumoniae in a neonatal intensive care unit in Zambia before and after an infection control bundle
Source: PLOS Glob Public Health. 2026 Feb 9;6(2):e0005965. doi: 10.1371/journal.pgph.0005965 (PMC12885268; doi:10.1371/journal.pgph.0005965)

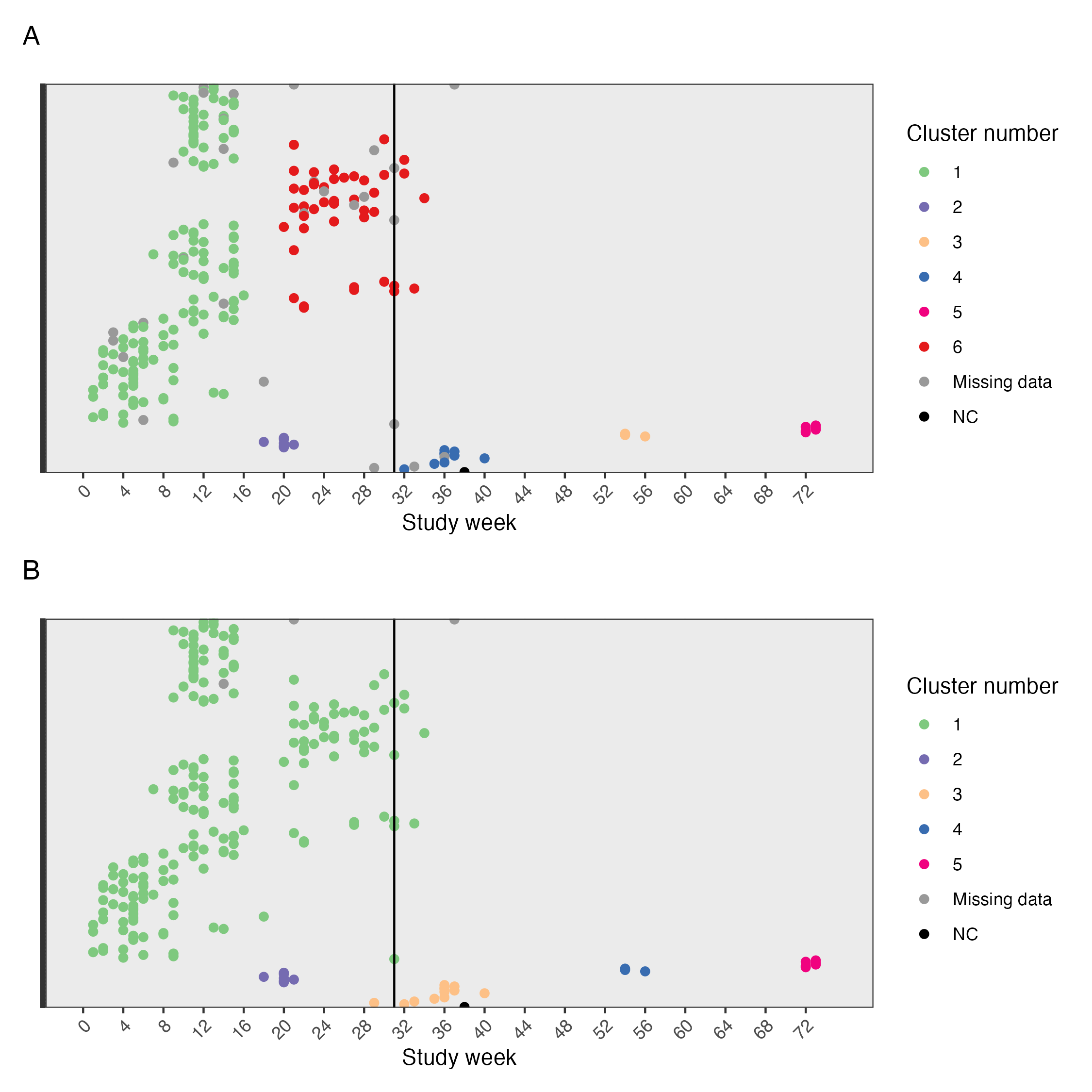

Supplement: S2 Fig — (A) Using culture date to define clusters if available, and admission date if not (n = 233), compared to (B) using only culture date (n = 206). (TIF) [file pgph.0005965.s004.tif]

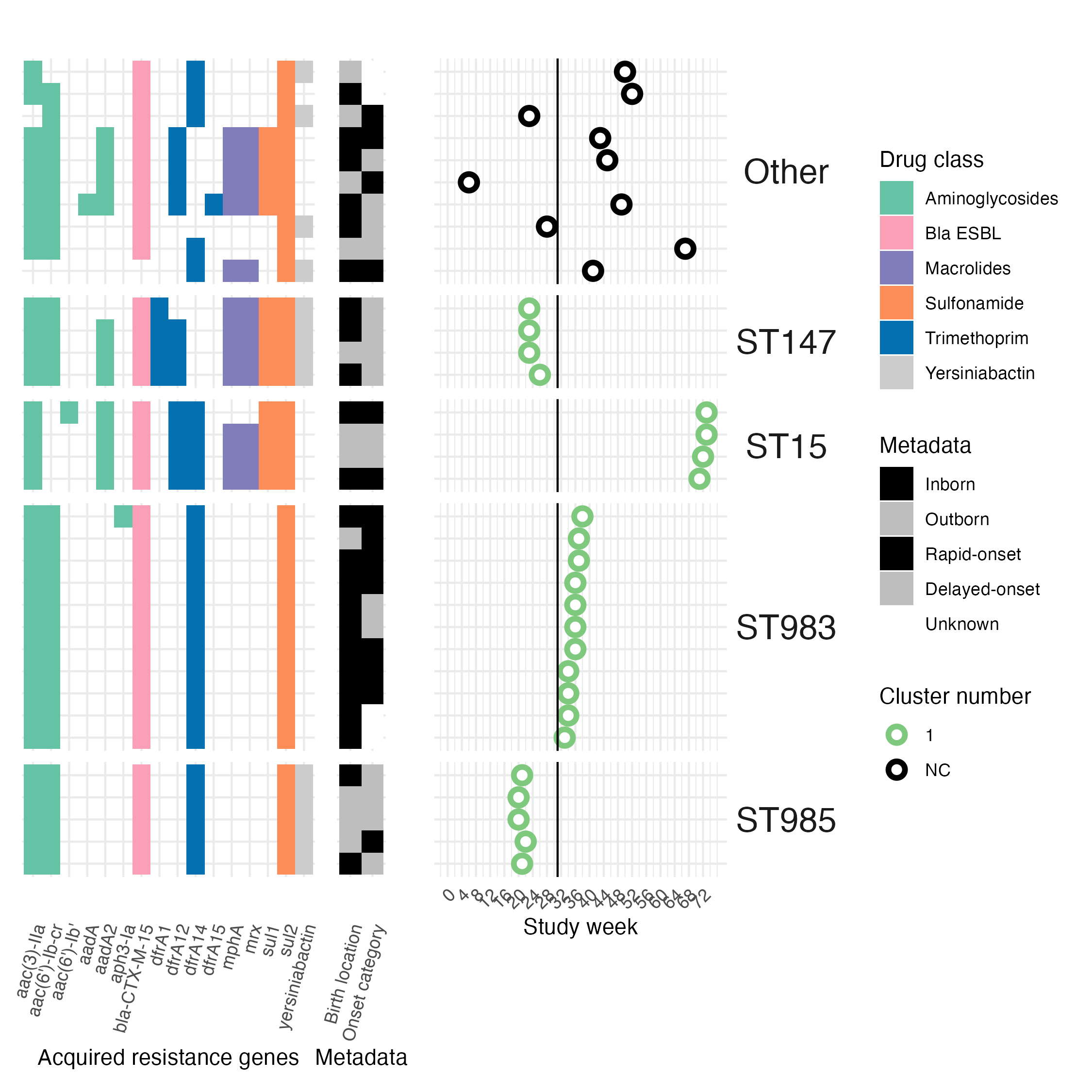

Supplement: S3 Fig — In all other STs for which a phylogenetic tree could not be constructed. The presence of an isolate in a cluster is indicated by the colour of the circle plotted by study week of infection. The solid vertical line represents the start of the implementation of the IPC bundle. (TIF) [file pgph.0005965.s005.tif]
