# Supplementary material for: Transmission dynamics of Klebsiella pneumoniae in a neonatal intensive care unit in Zambia before and after an infection control bundle
Source: PLOS Glob Public Health. 2026 Feb 9;6(2):e0005965. doi: 10.1371/journal.pgph.0005965 (PMC12885268; doi:10.1371/journal.pgph.0005965)
Supplement: S1 Appendix — The sensitivity of clustering to choice of SNV threshold (between 5–25) and temporal threshold (between 7–28 days) was assessed for all STs. (DOCX) [file pgph.0005965.s009.docx]

**Appendix S1. Clustering sensitivity analyses.**

The sensitivity of clustering to choice of SNV threshold (between 5–25) and temporal threshold (between 7–28 days) was assessed for all STs.

| ST307 | | **Temporal threshold (weeks)** | | | |
| --- | --- | --- | --- | --- | --- |
|  |  | **1** | **2** | **3** | **4** |
| **SNP distance threshold**  **5-20** | **No of clusters** | 8 | 7 | 5 | 5 |
|  | **No of clustered sequences** | 225/232 | 227/232 | 230/232 | 231/232 |
|  | **Median cluster size** | 7 | 7 | 7 | 7 |
|  | **Proportion of cases in a transmission cluster** | 97% | 98% | 99% | 100% |
|  | **Proportion of cases due to transmission** | 94% | 95% | 97% | 97% |

| ST101 | | **Temporal threshold (weeks)** | | | |
| --- | --- | --- | --- | --- | --- |
|  |  | **1** | **2** | **3** | **4** |
| **SNP distance threshold**  **5** | **No of clusters** | 1 | 2 | 2 | 2 |
|  | **No of clustered sequences** | 2/9 | 4/9 | 4/9 | 5/9 |
|  | **Median cluster size** | 2 | 2 | 2 | 2 |
|  | **Proportion of cases in a transmission cluster** | 22% | 44% | 44% | 56% |
|  | **Proportion of cases due to transmission** | 11% | 22% | 22% | 33% |
| **SNP distance threshold**  **10 - 25** | **No of clusters** | 1 | 2 | 2 | 2 |
|  | **No of clustered sequences** | 2/9 | 5/9 | 6/9 | 6/9 |
|  | **Median cluster size** | 2 | 2 | 3 | 3 |
|  | **Proportion of cases in a transmission cluster** | 22% | 56% | 67% | 67% |
|  | **Proportion of cases due to transmission** | 11% | 33% | 44% | 44% |

| ST2004 | | **Temporal threshold (weeks)** | | | |
| --- | --- | --- | --- | --- | --- |
|  |  | **1** | **2** | **3** | **4** |
| **SNP distance threshold**  **5-25** | **No of clusters** | 6 | 2 | 2 | 2 |
|  | **No of clustered sequences** | 44/47 | 47/47 | 47/47 | 47/47 |
|  | **Median cluster size** | 4 | 24 | 24 | 24 |
|  | **Proportion of cases in a transmission cluster** | 94% | 100% | 100% | 100% |
|  | **Proportion of cases due to transmission** | 81% | 96% | 96% | 96% |

| ST983 | | **Temporal threshold (weeks)** | | | |
| --- | --- | --- | --- | --- | --- |
|  |  | **1** | **2** | **3** | **4** |
| **SNP distance threshold**  **5-25** | **No of clusters** | 2 | 1 | 1 | 1 |
|  | **No of clustered sequences** | 9/9 | 9/9 | 9/9 | 9/9 |
|  | **Median cluster size** | 4 | 9 | 9 | 9 |
|  | **Proportion of cases in a transmission cluster** | 100% | 100% | 100% | 100% |
|  | **Proportion of cases due to transmission** | 78% | 89% | 89% | 89% |

| ST147 | | **Temporal threshold (weeks)** | | | |
| --- | --- | --- | --- | --- | --- |
|  |  | **1** | **2** | **3** | **4** |
| **SNP distance threshold**  **5-25** | **No of clusters** | 1 | 1 | 1 | 1 |
|  | **No of clustered sequences** | 3/4 | 3/4 | 4/4 | 4/4 |
|  | **Median cluster size** | 3 | 3 | 4 | 4 |
|  | **Proportion of cases in a transmission cluster** | 75% | 75% | 100% | 100% |
|  | **Proportion of cases due to transmission** | 50% | 50% | 75% | 75% |

| **SNP distance threshold**  **5-25** | ST15 | **Temporal threshold (weeks)** | ST985 | **Temporal threshold (weeks)** |
| --- | --- | --- | --- | --- |
|  |  | **1-4** |  | **1-4** |
|  | **No of clusters** | 1 | **No of clusters** | 1 |
|  | **No of clustered sequences** | 4/4 | **No of clustered sequences** | 5/5 |
|  | **Median cluster size** | 4 | **Median cluster size** | 5 |
|  | **Proportion of cases in a transmission cluster** | 100% | **Proportion of cases in a transmission cluster** | 100% |
|  | **Proportion of cases due to transmission** | 75% | **Proportion of cases due to transmission** | 80% |

| ST985 | | **Temporal threshold (weeks)** |
| --- | --- | --- |
|  |  | **1-4** |
| **SNP distance threshold**  **5-25** | **No of clusters** | 1 |
|  | **No of clustered sequences** | 5/5 |
|  | **Median cluster size** | 5 |
|  | **Proportion of cases in a transmission cluster** | 100 |
|  | **Proportion of cases due to transmission** | 80 |
