# Supplementary material for: Transmission dynamics of Klebsiella pneumoniae in a neonatal intensive care unit in Zambia before and after an infection control bundle
Source: PLOS Glob Public Health. 2026 Feb 9;6(2):e0005965. doi: 10.1371/journal.pgph.0005965 (PMC12885268; doi:10.1371/journal.pgph.0005965)
Supplement: Attachment — Submitted filename: Response to Reviewers.pdf [file pgph.0005965.s010.pdf]

Reviewer #1

This is a fascinating paper presenting a highly robust genomic analysis of a large collection of Klebsiella pneumoniae isolates. Although the study is limited by the absence of contemporaneous outbreak investigations and environmental sampling to more definitively delineate transmission pathways, the authors effectively triangulate genomic, temporal, and clinical data to generate plausible inferences regarding transmission dynamics and the hypothesized impact of the IPC bundle.

Minor Comments

1. The opening paragraph would benefit from clarification of denominators to avoid confusion. In the second sentence, I suggest explicitly adding "sepsis-related" to clarify the statistic, e.g. "93.9% of global sepsis-related neonatal deaths in 2019". As currently written, the transition between infectious neonatal deaths overall and neonatal sepsis specifically is potentially confusing.

Response: This has been modified to read:

Line 95-97 "Neonatal sepsis in LMICs contributes significantly to this high burden, accounting for 78.9% of the world's total reported **neonatal sepsis cases** and 93.9% of global neonatal **sepsis-related deaths** in 2019 (3-5)."

2. The statement that "this is the first study to perform genomic characterization using WGS, including sequence typing and AMR gene profiling, to explore the prevalence and distribution of STs" appears overly broad. Do the authors mean this is the first such study from Zambia?

Response: This has been modified to add "in Zambia", line 691.

3. The observation that the dominant outbreak clone (ST307) was predicted to be "capsule-null" is fascinating, particularly given that capsule-null K. pneumoniae are rarely recovered from blood due to the capsule's role in evading serum complement and phagocytosis. The authors reasonably speculate that these isolates may in fact have been encapsulated via an alternative locus, given the scale and persistence of invasive disease. However, an additional epidemiologic explanation deserves consideration: An alternative hypothesis is that these organisms may not have required particularly strong immune evasion traits because they were introduced directly into the bloodstream—for example via contaminated intravenous fluids, reagents, or equipment—rather than via a colonization-preceding translocation pathway. This interpretation aligns with later discussion of rapid-onset cases and would be consistent with sustained transmission from a persistent environmental reservoir rather than enhanced intrinsic virulence. The

authors go on to conclude that this finding “underscores the importance of considering both O- and K-antigen variability in the design of a maternal vaccine.” I agree, but would go further: these data also underscore the importance of real-time epidemiologic investigation and environmental source tracing, as this clone may not have been uniquely virulent but rather uniquely positioned to persist in, and be transmitted from, the NICU environment through breaches in medication safety or IPC. These clones, due to their high numbers, are likely making the “cut” for a maternal vaccine, but they may in fact not be very virulent, and may simply be reflective of their persistence in the environment of that NICU.

Response: The Discussion has been updated as follows:

Line 710-719 “Given the susceptibility of unencapsulated bacteria to host immune defences, it seems unlikely these isolates lacked a capsule yet maintained sustained transmission at the observed scale over a sustained period of time**, although it is conceivable if the bacteria were directly inoculated into the bloodstream via contaminated IV fluids, as has been reported in other LMIC NICU settings (42)**. This observation, alongside the dynamic shifts in K-type prevalence, underscores the importance of considering both O- and K-antigen variability in the design of a maternal vaccine, **and also the relevance of antibody-based immunity to bacterial strains directly introduced to the bloodstream via contaminated reagents or equipment**.”

4. The authors should clarify how repeat blood cultures from the same patient were handled. Were multiple cultures obtained per neonate, and if so, what criteria (time interval, clinical resolution) were used to define a new episode versus persistence of the same infection?

Response: This was mentioned in the Methods, however we have updated for clarity as follows (line 203):

“**Where multiple *K. pneumoniae* blood culture isolates were available from the same neonate**, only the first WGS-confirmed *K. pneumoniae* isolate recorded was included, thus excluding n=2 assemblies.”

5. Given the authors’ own acknowledgment that limited availability of blood cultures constrains accurate diagnosis and outbreak detection in LMIC NICUs, it would be helpful to explicitly state whether blood culture bottles were consistently available throughout the entire surveillance period. If access was uninterrupted, this should be clearly stated, as it strengthens confidence that observed temporal trends reflect true changes in transmission rather than diagnostic availability.

Response: We have added the following to the Methods section where culture methods are discussed:

Line 184 “Blood culture bottles, Vitek cards, and other microbiological reagents were funded and either locally procured or imported as part of the research study to ensure supply was uninterrupted during the study period.”

6. The authors’ discussion of the limitations of conventional HCAI definitions—particularly the EOS vs. LOS dichotomy and the routine attribution of EOS to vertical transmission—is a notable strength of this paper. The genomic evidence convincingly demonstrates that many “early-onset” cases are compatible with nosocomial transmission, challenging entrenched assumptions in neonatal epidemiology.

Response: Thank you for this comment

7. Please spell out “IV” as “intravenous” at first mention.

Response: done (line 652)

8. The paper does an admirable job of interrogating transmission pathways and the impact of the IPC bundle using genomic data and limited clinical data alone. The overall reduction in bloodstream infections is compelling. However, one unavoidable limitation is the possibility that the pre-intervention period coincided with an anomalously large outbreak. While the authors appropriately acknowledge this indirectly, readers will naturally wonder whether reduced transmission was sustained beyond the study period. Understanding that this is beyond the scope of this analysis, the authors attempted to look at the bundle’s efficacy from a different angle: by examining rapid- versus delayed-onset infections, hypothesizing that a decolonization-focused bundle would preferentially reduce delayed-onset sepsis. They write, “3 out of 6 clusters post-implementation included at least half rapid-onset infections”, which is factually correct, but the aggregate data (Table 2) show that 44% (82/187) were rapid onset in the baseline period, and 37% (28/76) were rapid onset in the post-implementation period. One might have expected a decline in delayed-onset cases if decolonization was the dominant mechanism of effect and the aggregate data don't reflect this. This discrepancy does not undermine the findings, but it does suggest that the observed benefit may reflect a broader unit-wide IPC ‘push’ rather than the specific mechanistic effects of individual bundle components. This distinction is important, as it informs how future IPC bundles should be designed and evaluated. It may bear mentioning, given the sheer magnitude of rapid-onset infections in this analysis, that future IPC bundles

should explicitly incorporate interventions aimed at preventing rapid-onset sepsis, including strengthened medication safety, IV fluid handling, and surveillance for contaminated reagents. These elements were not clearly part of the intervention described here (unless embedded within IPC training), and their omission may explain why rapid-onset transmission persisted despite overall gains.

Response: The concluding paragraph has been modified to include the following:

Line 828 “Furthermore the high rate of rapid-onset infections observed in this study, both pre- and post-implementation of the IPC bundle, underscores the critical importance of explicitly incorporating measures to strengthen medication safety, IV fluid handling, and surveillance for contaminated reagents, as part of IPC actions.”

Reviewer #2

Phillips et al. present a compelling study investigating whole-genome sequencing (WGS) data from over 400 Klebsiella pneumoniae isolates collected from neonates at a large tertiary care hospital in Zambia. The WGS data derive from a longitudinal study design consisting of a 6-month baseline period, followed by a 6-week infection prevention and control (IPC) intervention and an 11-month post-intervention assessment. Genomic analysis revealed that nearly all isolates carried extended-spectrum β-lactamase (ESBL) genes and belonged to the globally disseminated ST307 lineage. Further analyses demonstrated that ST307 was responsible for widespread transmission during the baseline period and that IPC measures effectively halted circulation of this outbreak lineage. WGS also identified the emergence of a genetically distinct ST307 strain post-intervention, suggesting clearance of the original strain and subsequent introduction of a new one. Similar transmission dynamics were observed in other major lineages, underscoring the effectiveness of IPC practices in interrupting outbreaks. Notably, baseline ST307 isolates exhibited disruption of the KL102 locus, raising the possibility that capsule alteration may have conferred an in-host advantage, such as enhanced biofilm formation. Overall, this study provides a strong example of how integrated IPC strategies and WGS surveillance can be used to intervene on high-risk pathogens. The bioinformatic methods are sound, and the conclusions are well supported by the results. Therefore, only a few minor edits are recommended.

Minor Comments

1. Please add scale bars to all phylogenetic trees to facilitate interpretation of genetic distances.

Response: Figures with trees (1, 4, S6) have been updated to include scale bars.

2. Consider moving lines 746–747 from the Discussion section to line 315 in the Results section, as this content appears more appropriate for reporting primary findings.

Response: The line in question is “A small number of isolates carried a bla_{NDM-5} carbapenemase gene, belonging to ST340 (n=2) and ST5856 (n=9).” The numbers (n=2, n=9) have now been added to the relevant section in Results, which now reads:

Line 329 “Few isolates had an acquired carbapenemase gene (n=11/411, 2.7%; all ST5856 **(n=9)** or ST340 **(n=2)** carrying bla_{NDM-5}).”
